# Supplementary material for: Alternative splicing in seasonal plasticity and the potential for adaptation to environmental change
Source: Nat Commun. 2022 Feb 8;13:755. doi: 10.1038/s41467-022-28306-8 (PMC8825856; doi:10.1038/s41467-022-28306-8)
Supplement: Supplementary file 1 — Supplementary Information [file 41467_2022_28306_MOESM1_ESM.docx]

Supplementary Information

Alternative splicing in seasonal plasticity and the potential for adaptation to environmental change

RA Steward, MA de Jong, V Oostra, CW Wheat

Table of Contents

[Supplementary Tables 2](#_Toc93322478)

[Supplementary Table 1 2](#_Toc93322479)

[Supplementary Table 2 2](#_Toc93322480)

[Supplementary Table 3 3](#_Toc93322481)

[Supplementary Table 4. 4](#_Toc93322482)

[Supplementary Table 5. 5](#_Toc93322483)

[Supplementary Table 6. 6](#_Toc93322484)

[Supplementary Table 7 7](#_Toc93322485)

[Supplementary Table 8. 8](#_Toc93322486)

[Supplementary Table 9 9](#_Toc93322487)

[Supplementary Figures 10](#_Toc93322488)

[Supplementary Fig. 1 10](#_Toc93322489)

[Supplementary Fig. 2 11](#_Toc93322490)

[Supplementary Fig. 3 12](#_Toc93322491)

[Supplementary Fig. 4 15](#_Toc93322492)

[Supplementary Fig. 5 17](#_Toc93322493)

[Supplementary Fig. 6 18](#_Toc93322494)

[Supplementary Fig. 7 19](#_Toc93322495)

[Supplementary Fig. 8 20](#_Toc93322496)

[Supplementary Fig. 9 21](#_Toc93322497)

[Supplementary Fig. 10 22](#_Toc93322498)

[Supplementary Fig. 11 23](#_Toc93322499)

[Supplementary Fig. 12 24](#_Toc93322500)

[Supplementary Fig. 13 25](#_Toc93322501)

[Supplementary Fig. 14 26](#_Toc93322502)

[Supplementary Notes 27](#_Toc93322503)

[Supplementary Note 1. 27](#_Toc93322504)

# Supplementary Tables

Supplementary Table 1. Sample sizes by family and seasonal morph.

| **Family** | **Abdomen** | | **Thorax** | |
| --- | --- | --- | --- | --- |
|  | **Dry** | **Wet** | **Dry** | **Wet** |
| 21 | 4 | 4 | 4 | 4 |
| 29 | 4 | 6 | 6 | 6 |
| 30 | 6 | 6 | 6 | 6 |
| 37 | 6 | 6 | 6 | 6 |
| 53 | 4 | 3 | 4 | 4 |
| 60 | 6 | 6 | 5 | 5 |
| 63 | 4 | 4 | 4 | 4 |
| Total | 34 | 35 | 35 | 35 |

Supplementary Table 2. edgeR analysis of exon-level alternative splicing between tissues and by season, family and season x family interaction (SxF). Read counts were quantified at the feature level (exon, 196,681 in annotation) using featureCounts (n = 118,950 assigned). Abdomen and thorax samples were filtered and analyzed separately. edgeR was used to compare exon expression between dry and wet morphs. Genes with differentially expressed exons (normalized by average exon expression within genes) were identified as differentially spliced (DS) using the diffspliceDGE function (Simes correction of quasi-likelihood F-test). P-values were corrected across all tests within tissue using the Benjamini-Hochberg method.

| **edgeR Differential Splicing** | **Abdomen vs. thorax** | **Abdomen: dry vs. wet** | | | **Thorax: dry vs. wet** | | |
| --- | --- | --- | --- | --- | --- | --- | --- |
| Filtered exons | 64832 | 69005 | | | 46289 | | |
| genes with only one exon | 1866 | 1890 | | | 2035 | | |
| Multi-exon genes | 8533 | 8880 | | | 6684 | | |
| Exons in multi-exon genes | 62966 | 67115 | | | 44254 | | |
| Mean exons in a gene | 6 | 6 | | | 5 | | |
| Maximum exons in a gene | 136 | 108 | | | 135 | | |
|  | **---** | **Season** | **Family** | **SxF** | **Season** | **Family** | **SxF** |
| DS genes (p < 0.05) | 4697 | 799 | 2697 | 496 | 347 | 1438 | 328 |
| DS genes, corrected (adj. p < 0.05) | 4327 | 363 | 1170 | 51 | 172 | 513 | 16 |
| Percent of filtered genes that are DS (adj. p < 0.05) | 50.7% | 4.1% | 13.2% | 0.6% | 2.6% | 7.7% | 0.3% |
| Exons in corrected DS genes | 41514 | 4309 | 12931 | 885 | 2489 | 5561 | 494 |
| DE exons in DS genes | 21376 | 347 | 5759 | 198 | 659 | 2239 | 90 |
| Percent of exons in DS genes that are DE | 51.5% | 8.0% | 44.5% | 22.4% | 26.5% | 40.3% | 18.2% |

Supplementary Table 3. edgeR analysis of gene-level differential expression by season, family and season x family interaction (SxF). Read counts were quantified at the meta-feature level (geneid, n = 15,845 in annotation) using featureCounts (RSubread). Abdomen and thorax samples were filtered and analyzed separately. Counts were filtered using the filterByExpr function in the edgeR package. Differential gene expression was analyzed for all filtered using quasi-likelihood F-tests. We then identified the subset of filtered genes with more than one exon (multi-exon genes) that corresponded with genes that might undergo alternative splicing. P-values were corrected across all tests within tissue using the Benjamini-Hochberg method.

| **edgeR Differential Expression** | **Abdomen vs. thorax** | **Abdomen: dry vs. wet** | | | **Thorax: dry vs. wet** | | |
| --- | --- | --- | --- | --- | --- | --- | --- |
| Total filtered genes | 11021 | 11364 | | | 9516 | | |
| Filtered multi-exon genes | 10214 | 10523 | | | 8835 | | |
|  | **---** | **Season** | **Family** | **SxF** | **Season** | **Family** | **SxF** |
| DE genes (p < 0.05) | 9960 | 6386 | 8815 | 699 | 5878 | 5163 | 735 |
| DE genes, corrected (adj. p < 0.05) | 9942 | 5744 | 8546 | 9 | 5509 | 4370 | 26 |
| Percent of genes that are DE (adj. p < 0.05) | 94.9% | 50.6% | 75.2% | 0.1% | 57.9% | 45.9% | 0.3% |
| DE multi-exon genes (p < 0.05) | 9234 | 5928 | 8219 | 656 | 5455 | 4807 | 686 |
| DE multi-exon genes, corrected (adj. p < 0.05) | 9218 | 5335 | 7970 | 9 | 5111 | 4075 | 25 |
| Percent of multi-exon genes that are DE (adj. p < 0.05) | 90.3% | 50.7% | 75.7% | 0.1% | 57.9% | 46.1% | 0.3% |

Supplementary Table 4. One-sided Fisher’s exact tests were used to test the significance of overlapping gene sets identified as differentially spliced or expressed by edgeR analyses. P-values were adjusted for 66 tests using the Benjamini-Hochberg correction. Gene sets sizes were based on the total number of unique gene IDs tested in both analyses.

| **Comparison** | **Tissue** | **G1** | **G2** | **Multi-exon genes** | **N1** | **N2** | **G1 only** | **G2 only** | **Actual overlap** | **Expected overlap** | **RF** | **Set size** | **Odds ratio** | **P-value** | **Adj. P-value** | **Sig.** |
| --- | --- | --- | --- | --- | --- | --- | --- | --- | --- | --- | --- | --- | --- | --- | --- | --- |
| Effect within abdomen (Fig. 1B) | Abdomen | DE Season | DE Family | 10523 | 5335 | 7970 | 1169 | 3804 | 4166 | 4040.7 | 1.03 | 2553 | 1.30 | 6.74E-09 | 1.00E-07 | ** |
| Effect within abdomen (Fig. 1B) | Abdomen | DE Season | DE SxF | 10523 | 5335 | 9 | 5327 | 1 | 8 | 4.6 | 1.75 | 10514 | 7.79 | 2.15E-02 | 6.46E-02 |  |
| Effect within abdomen (Fig. 1B) | Abdomen | DE Family | DE SxF | 10523 | 7970 | 9 | 7963 | 2 | 7 | 6.8 | 1.03 | 10514 | 1.12 | 6.21E-01 | 6.21E-01 |  |
| Effect within thorax (Fig. 1B) | Thorax | DE Season | DE Family | 8835 | 5111 | 4075 | 2691 | 1655 | 2420 | 2357.4 | 1.03 | 4760 | 1.12 | 3.61E-03 | 1.45E-02 | * |
| Effect within thorax (Fig. 1B) | Thorax | DE Season | DE SxF | 8835 | 5111 | 25 | 5094 | 8 | 17 | 14.5 | 1.18 | 8810 | 1.55 | 2.06E-01 | 4.11E-01 |  |
| Effect within thorax (Fig. 1B) | Thorax | DE Family | DE SxF | 8835 | 4075 | 25 | 4053 | 3 | 22 | 11.5 | 1.91 | 8810 | 8.61 | 1.59E-05 | 1.11E-04 | *** |
| Effect within abdomen (Fig. 1A) | Abdomen | DS Season | DS Family | 8880 | 366 | 1168 | 217 | 1019 | 149 | 48.1 | 3.10 | 7712 | 5.05 | 1.90E-41 | 3.05E-40 | *** |
| Effect within abdomen (Fig. 1A) | Abdomen | DS Season | DS SxF | 8880 | 366 | 51 | 352 | 1019 | 14 | 2.1 | 6.66 | 8829 | 9.11 | 1.03E-08 | 8.22E-08 | *** |
| Effect within abdomen (Fig. 1A) | Abdomen | DS Family | DS SxF | 8880 | 1168 | 51 | 1125 | 8 | 43 | 6.7 | 6.41 | 8829 | 36.78 | 1.46E-30 | 2.05E-29 | *** |
| Effect within thorax (Fig. 1A) | Thorax | DS Season | DS Family | 6684 | 172 | 513 | 103 | 444 | 69 | 13.2 | 5.23 | 6171 | 8.67 | 1.16E-31 | 1.74E-30 | *** |
| Effect within thorax (Fig. 1A) | Thorax | DS Season | DS SxF | 6684 | 172 | 16 | 164 | 8 | 8 | 0.4 | 19.4 | 6668 | 39.52 | 1.77E-09 | 1.94E-08 | *** |
| Effect within thorax (Fig. 1A) | Thorax | DS Family | DS SxF | 6684 | 513 | 16 | 497 | 0 | 16 | 1.2 | 13.0 | 6668 | Inf | 1.17E-18 | 1.52E-17 | *** |
| DE vs. DS within abdomen (Fig. 2A) | Abdomen | DE Season | DS Season | 10523 | 5335 | 366 | 5117 | 148 | 218 | 185.6 | 1.18 | 10157 | 1.45 | 3.25E-04 | 1.95E-03 | ** |
| DE vs. DS within abdomen (Fig. 2A) | Abdomen | DE Family | DS Family | 10523 | 7970 | 1168 | 7007 | 205 | 963 | 884.6 | 1.10 | 9355 | 1.57 | 3.08E-09 | 3.08E-08 | *** |
| DE vs. DS within abdomen (Fig. 2A) | Abdomen | DE SxF | DS SxF | 10523 | 9 | 51 | 9 | 51 | 0 | 0 | NA | 10472 | NA | NA | NA |  |
| DE vs. DS within thorax (Fig. S2A) | Thorax | DE Season | DS Season | 8835 | 5111 | 172 | 4993 | 54 | 118 | 99.5 | 1.19 | 8663 | 1.61 | 2.22E-03 | 1.11E-02 | ** |
| DE vs. DS within thorax (Fig. S2A) | Thorax | DE Family | DS Family | 8835 | 4075 | 513 | 3762 | 200 | 313 | 236.6 | 1.3 | 8322 | 1.90 | 2.20E-12 | 2.65E-11 | * |
| DE vs. DS within thorax (Fig. S2A) | Thorax | DE SxF | DS SxF | 8835 | 25 | 16 | 25 | 16 | 0 | 0.04 | NA | 8819 | NA | NA | NA |  |

Supplementary Table 5. Tukey’s HSD multiple comparisons (two-sided) of nucleotide diversity (π) between groups of DS and DE genes in the abdomen and thorax. P-values were adjusted for 30 tests. Likely because of the conservatism of the p-value adjustment, significant differences were found for only a subset of the meaningful differences identified by the Bayesian linear model (* in the hyp.test column).

| **Tissue** | **Effect** | **G1** | **G2** | **Estimate** | **LCL^1^** | **HCL^2^** | **Adj. P-value** | **Sig.** | **Hyp. test** |
| --- | --- | --- | --- | --- | --- | --- | --- | --- | --- |
| Abdomen | DEDS_F | not DE or DS | DS only | 2.55E-04 | -7.71E-04 | 1.28E-03 | 9.19E-01 | ns |  |
| Abdomen | DEDS_F | not DE or DS | DE only | 9.04E-04 | 5.96E-04 | 1.21E-03 | 0.00E+00 | **** | * |
| Abdomen | DEDS_F | not DE or DS | DE and DS | 1.47E-03 | 9.48E-04 | 1.99E-03 | 0.00E+00 | **** | * |
| Abdomen | DEDS_F | DS only | DE only | 6.48E-04 | -3.67E-04 | 1.66E-03 | 3.56E-01 | ns |  |
| Abdomen | DEDS_F | DS only | DE and DS | 1.21E-03 | 1.15E-04 | 2.31E-03 | 2.35E-02 | * | * |
| Abdomen | DEDS_F | DE only | DE and DS | 5.66E-04 | 6.56E-05 | 1.07E-03 | 1.92E-02 | * | * |
| Abdomen | DEDS_S | not DE or DS | DS only | -9.65E-04 | -2.16E-03 | 2.30E-04 | 1.61E-01 | ns | * |
| Abdomen | DEDS_S | not DE or DS | DE only | 3.73E-04 | 8.25E-05 | 6.64E-04 | 5.40E-03 | ** | * |
| Abdomen | DEDS_S | not DE or DS | DE and DS | -7.59E-04 | -1.75E-03 | 2.36E-04 | 2.03E-01 | ns |  |
| Abdomen | DEDS_S | DS only | DE only | 1.34E-03 | 1.39E-04 | 2.54E-03 | 2.16E-02 | * | * |
| Abdomen | DEDS_S | DS only | DE and DS | 2.06E-04 | -1.32E-03 | 1.74E-03 | 9.86E-01 | ns |  |
| Abdomen | DEDS_S | DE only | DE and DS | -1.13E-03 | -2.13E-03 | -1.32E-04 | 1.91E-02 | * | * |
| Abdomen | DEDS_SxF | not DE or DS | DS only | -1.21E-04 | -1.95E-03 | 1.71E-03 | 9.87E-01 | ns |  |
| Abdomen | DEDS_SxF | not DE or DS | DE only | -3.78E-04 | -6.20E-03 | 5.44E-03 | 9.87E-01 | ns |  |
| Abdomen | DEDS_SxF | DS only | DE only | -2.56E-04 | -6.35E-03 | 5.84E-03 | 9.95E-01 | ns |  |
| Thorax | DEDS_F | not DE or DS | DS only | 3.33E-04 | -6.70E-04 | 1.34E-03 | 8.29E-01 | ns |  |
| Thorax | DEDS_F | not DE or DS | DE only | 7.00E-04 | 3.73E-04 | 1.03E-03 | 2.00E-07 | **** | * |
| Thorax | DEDS_F | not DE or DS | DE and DS | 1.72E-03 | 9.07E-04 | 2.54E-03 | 4.00E-07 | **** | * |
| Thorax | DEDS_F | DS only | DE only | 3.67E-04 | -6.53E-04 | 1.39E-03 | 7.92E-01 | ns |  |
| Thorax | DEDS_F | DS only | DE and DS | 1.39E-03 | 1.26E-04 | 2.66E-03 | 2.45E-02 | * | * |
| Thorax | DEDS_F | DE only | DE and DS | 1.02E-03 | 1.87E-04 | 1.86E-03 | 9.14E-03 | ** | * |
| Thorax | DEDS_S | not DE or DS | DS only | -1.19E-03 | -3.12E-03 | 7.32E-04 | 3.83E-01 | ns |  |
| Thorax | DEDS_S | not DE or DS | DE only | -2.15E-04 | -5.20E-04 | 8.98E-05 | 2.68E-01 | ns |  |
| Thorax | DEDS_S | not DE or DS | DE and DS | -1.20E-03 | -2.50E-03 | 9.40E-05 | 8.03E-02 | ns | * |
| Thorax | DEDS_S | DS only | DE only | 9.78E-04 | -9.49E-04 | 2.90E-03 | 5.60E-01 | ns |  |
| Thorax | DEDS_S | DS only | DE and DS | -1.19E-05 | -2.31E-03 | 2.29E-03 | 1.00E+00 | ns |  |
| Thorax | DEDS_S | DE only | DE and DS | -9.90E-04 | -2.29E-03 | 3.12E-04 | 2.06E-01 | ns | * |
| Thorax | DEDS_SxF | not DE or DS | DS only | 3.09E-04 | -2.87E-03 | 3.49E-03 | 9.72E-01 | ns |  |
| Thorax | DEDS_SxF | not DE or DS | DE only | 8.04E-04 | -2.20E-03 | 3.80E-03 | 8.05E-01 | ns |  |
| Thorax | DEDS_SxF | DS only | DE only | 4.95E-04 | -3.87E-03 | 4.86E-03 | 9.62E-01 | ns |  |

*^1^Lower 95% confidence limit*

*^2^Upper 95% confidence limit*

Supplementary Table 6. Dunn’s test multiple comparisons (two-sided) of nucleotide divergence (ω) between groups of DS and DE genes in the abdomen and thorax. P-values were adjusted for 30 tests. Results of meaningful differences identified by the Bayesian linear model (* in the hyp.test column) are also indicated.

| **Tissue** | **Effect** | **G1** | **G2** | **N1** | **N2** | **Statistic** | **P-value** | **Adj. P-value** | **Sig.** | **Hyp. test** |
| --- | --- | --- | --- | --- | --- | --- | --- | --- | --- | --- |
| Abdomen | DEDS_F | not DE or DS | DS only | 1492 | 148 | 0.11 | 9.13E-01 | 9.68E-01 | ns |  |
| Abdomen | DEDS_F | not DE or DS | DE only | 1492 | 4115 | 3.22 | 1.27E-03 | 7.64E-03 | ** | * |
| Abdomen | DEDS_F | not DE or DS | DE and DS | 1492 | 614 | 0.12 | 9.03E-01 | 9.68E-01 | ns |  |
| Abdomen | DEDS_F | DS only | DE only | 148 | 4115 | 1.05 | 2.93E-01 | 5.87E-01 | ns |  |
| Abdomen | DEDS_F | DS only | DE and DS | 148 | 614 | -0.04 | 9.68E-01 | 9.68E-01 | ns |  |
| Abdomen | DEDS_F | DE only | DE and DS | 4115 | 614 | -2.12 | 3.44E-02 | 1.03E-01 | ns | * |
| Abdomen | DEDS_S | not DE or DS | DS only | 2955 | 113 | 0.16 | 8.77E-01 | 9.34E-01 | ns |  |
| Abdomen | DEDS_S | not DE or DS | DE only | 2955 | 3140 | 0.27 | 7.87E-01 | 9.34E-01 | ns |  |
| Abdomen | DEDS_S | not DE or DS | DE and DS | 2955 | 161 | 1.38 | 1.67E-01 | 5.81E-01 | ns |  |
| Abdomen | DEDS_S | DS only | DE only | 113 | 3140 | -0.08 | 9.34E-01 | 9.34E-01 | ns |  |
| Abdomen | DEDS_S | DS only | DE and DS | 113 | 161 | 0.79 | 4.29E-01 | 8.58E-01 | ns |  |
| Abdomen | DEDS_S | DE only | DE and DS | 3140 | 161 | 1.30 | 1.94E-01 | 5.81E-01 | ns |  |
| Abdomen | DEDS_SxF | not DE or DS | DS only | 6331 | 35 | -0.20 | 8.45E-01 | 9.87E-01 | ns |  |
| Abdomen | DEDS_SxF | not DE or DS | DE only | 6331 | 3 | -0.04 | 9.67E-01 | 9.87E-01 | ns |  |
| Abdomen | DEDS_SxF | DS only | DE only | 35 | 3 | 0.02 | 9.87E-01 | 9.87E-01 | ns |  |
| Thorax | DEDS_F | not DE or DS | DS only | 4013 | 158 | -0.92 | 3.58E-01 | 6.64E-01 | ns |  |
| Thorax | DEDS_F | not DE or DS | DE only | 4013 | 2062 | 0.77 | 4.42E-01 | 6.64E-01 | ns |  |
| Thorax | DEDS_F | not DE or DS | DE and DS | 4013 | 223 | 0.34 | 7.31E-01 | 8.77E-01 | ns |  |
| Thorax | DEDS_F | DS only | DE only | 158 | 2062 | 1.16 | 2.48E-01 | 6.64E-01 | ns |  |
| Thorax | DEDS_F | DS only | DE and DS | 158 | 223 | 0.94 | 3.45E-01 | 6.64E-01 | ns |  |
| Thorax | DEDS_F | DE only | DE and DS | 2062 | 223 | 0.04 | 9.68E-01 | 9.68E-01 | ns |  |
| Thorax | DEDS_S | not DE or DS | DS only | 3274 | 41 | 0.10 | 9.19E-01 | 9.19E-01 | ns |  |
| Thorax | DEDS_S | not DE or DS | DE only | 3274 | 3045 | -2.99 | 2.75E-03 | 1.65E-02 | * | * |
| Thorax | DEDS_S | not DE or DS | DE and DS | 3274 | 96 | -0.30 | 7.61E-01 | 9.19E-01 | ns |  |
| Thorax | DEDS_S | DS only | DE only | 41 | 3045 | -0.58 | 5.61E-01 | 9.19E-01 | ns |  |
| Thorax | DEDS_S | DS only | DE and DS | 41 | 96 | -0.25 | 7.99E-01 | 9.19E-01 | ns |  |
| Thorax | DEDS_S | DE only | DE and DS | 3045 | 96 | 0.42 | 6.72E-01 | 9.19E-01 | ns |  |
| Thorax | DEDS_SxF | not DE or DS | DS only | 6432 | 12 | -0.29 | 7.71E-01 | 7.71E-01 | ns |  |
| Thorax | DEDS_SxF | not DE or DS | DE only | 6432 | 12 | 0.31 | 7.59E-01 | 7.71E-01 | ns |  |
| Thorax | DEDS_SxF | DS only | DE only | 12 | 12 | 0.42 | 6.72E-01 | 7.71E-01 | ns |  |

Supplementary Table 7. rMATS analysis of event-based alternative splicing between seasonal morphs. rMATS results were filtered to exclude events with support (>5 reads) in fewer than three individuals in one of the two seasonal morphs. Events with p-vlaues < 0.05 and DPSI (percent spliced in) > 0.05 were considered significantly differentially spliced (sig. events, see supplementary table 12). Expected significant events (exp. sig. events) were calculated as the proportion of an event type out of all detected events, multiplied by the total number of differentially spliced events for that type. Deviations from this expectation were evaluated using Fisher’s exact tests (two-sided). ORFs were considered to be maintained when the inclusion form was divisible by three.

| **Splice type** | **Genes** | **Events** | **Events/ gene** | **Sig. events** | **% Sig. events** | **Exp. Sig. Events** | **Adj. p-value** | **Events maintaining ORF** | **% events maintaining ORF** | **Sig. events maintaining ORF** | **% Sig. events maintaining ORF** |
| --- | --- | --- | --- | --- | --- | --- | --- | --- | --- | --- | --- |
| **Abdomen: dry vs. wet** | | | | | | | | | | | |
| A3SS | 1143 | 1983 | 1.73 | 128 | 6.5% | 215 | 5.25E-06 | 1316 | 66.4% | 109 | 85.2% |
| A5SS | 1209 | 2234 | 1.85 | 188 | 8.4% | 242 | 3.56E-02 | 1554 | 69.6% | 143 | 76.1% |
| SE | 1295 | 4715 | 3.64 | 826 | 17.5% | 511 | 6.60E-20 | 3939 | 83.5% | 715 | 86.6% |
| IR | 802 | 1120 | 1.40 | 74 | 6.6% | 121 | 2.69E-03 | 807 | 72.1% | 54 | 73.0% |
| MXE | 2113 | 6645 | 3.14 | 593 | 8.9% | 720 | 1.23E-03 | 5742 | 86.4% | 507 | 85.5% |
| Total | 6562 | 16697 | 2.54 | 1809 | 10.8% | -- | -- | 13358 | 80.0% | 1528 | 84.5% |
| **Thorax: dry vs. wet** | | | | | | | | | | | |
| A3SS | 556 | 1196 | 2.15 | 88 | 7.4% | 150 | 1.41E-04 | 800 | 66.9% | 76 | 86.4% |
| A5SS | 614 | 1491 | 2.43 | 118 | 7.9% | 186 | 2.36E-04 | 1040 | 69.8% | 90 | 76.3% |
| SE | 808 | 3762 | 4.66 | 644 | 17.1% | 471 | 1.14E-07 | 3060 | 81.3% | 524 | 81.4% |
| IR | 384 | 563 | 1.47 | 43 | 7.6% | 70 | 4.84E-02 | 409 | 72.6% | 35 | 81.4% |
| MXE | 1103 | 6579 | 5.96 | 807 | 12.3% | 823 | 1.00E+00 | 5509 | 83.7% | 692 | 85.7% |
| Total | 3465 | 13591 | 3.92 | 1700 | 12.5% | -- | -- | 10818 | 79.6% | 1417 | 83.4% |

Supplementary Table 8. Tukey’s HSD multiple comparisons (two-sided) of nucleotide diversity (π) between groups of genes (G1 and G2) containing splice events identified by rMATS. Nucleotide diversity was compared between genes without splicing, those with nonsignificant splice events and those with significantly different splice events between seasons. Diversity was also compared among spliced genes containing different splice events: A3SS, A5SS, SE, IR, and MXE.

| **Analysis** | **Tissue** | **G1** | **G2** | **Estimate** | **LCL^1^** | **HCL^2^** | **Adj. P-value** | **Sig.** | **Hyp. test** |
| --- | --- | --- | --- | --- | --- | --- | --- | --- | --- |
| Between None, AS and DS genes | Abdomen | None | AS | -4.15E-05 | -3.35E-04 | 2.52E-04 | 9.41E-01 | ns |  |
| Between None, AS and DS genes | Abdomen | None | DS | -1.01E-03 | -1.54E-03 | -4.80E-04 | 2.44E-05 | **** | * |
| Between None, AS and DS genes | Abdomen | AS | DS | -9.70E-04 | -1.52E-03 | -4.20E-04 | 1.05E-04 | *** | * |
| Between None, AS and DS genes | Thorax | None | AS | -4.69E-04 | -8.24E-04 | -1.14E-04 | 5.57E-03 | ** | * |
| Between None, AS and DS genes | Thorax | None | DS | -1.58E-03 | -2.24E-03 | -9.30E-04 | 0.00E+00 | **** | * |
| Between None, AS and DS genes | Thorax | AS | DS | -1.12E-03 | -1.81E-03 | -4.18E-04 | 5.27E-04 | *** | * |
| Between event types | Abdomen | A3SS | A5SS | -4.69E-04 | -1.76E-03 | 8.20E-04 | 9.06E-01 | ns | ns |
| Between event types | Abdomen | A3SS | SE | -3.00E-04 | -1.61E-03 | 1.01E-03 | 9.87E-01 | ns | ns |
| Between event types | Abdomen | A3SS | IR | -1.70E-03 | -3.17E-03 | -2.18E-04 | 1.37E-02 | * | * |
| Between event types | Abdomen | A3SS | MXE | 8.81E-04 | -2.08E-04 | 1.97E-03 | 1.91E-01 | ns | * |
| Between event types | Abdomen | A3SS | None | 1.76E-04 | -7.71E-04 | 1.12E-03 | 9.95E-01 | ns | ns |
| Between event types | Abdomen | A5SS | SE | 1.68E-04 | -1.14E-03 | 1.48E-03 | 9.99E-01 | ns | ns |
| Between event types | Abdomen | A5SS | IR | -1.23E-03 | -2.70E-03 | 2.46E-04 | 1.65E-01 | ns | * |
| Between event types | Abdomen | A5SS | MXE | 1.35E-03 | 2.67E-04 | 2.43E-03 | 5.16E-03 | ** | * |
| Between event types | Abdomen | A5SS | None | 6.45E-04 | -2.96E-04 | 1.59E-03 | 3.69E-01 | ns | ns |
| Between event types | Abdomen | SE | IR | -1.40E-03 | -2.89E-03 | 9.97E-05 | 8.35E-02 | ns | * |
| Between event types | Abdomen | SE | MXE | 1.18E-03 | 6.92E-05 | 2.29E-03 | 2.98E-02 | * | * |
| Between event types | Abdomen | SE | None | 4.77E-04 | -4.97E-04 | 1.45E-03 | 7.30E-01 | ns | ns |
| Between event types | Abdomen | IR | MXE | 2.58E-03 | 1.27E-03 | 3.88E-03 | 3.00E-07 | **** | * |
| Between event types | Abdomen | IR | None | 1.87E-03 | 6.85E-04 | 3.06E-03 | 1.03E-04 | *** | * |
| Between event types | Abdomen | MXE | None | -7.05E-04 | -1.34E-03 | -6.46E-05 | 2.11E-02 | * | * |
| Between event types | Thorax | A3SS | A5SS | 4.89E-04 | -1.18E-03 | 2.16E-03 | 9.61E-01 | ns | ns |
| Between event types | Thorax | A3SS | SE | 4.14E-04 | -1.13E-03 | 1.96E-03 | 9.73E-01 | ns | ns |
| Between event types | Thorax | A3SS | IR | -1.12E-03 | -3.13E-03 | 8.91E-04 | 6.06E-01 | ns | ns |
| Between event types | Thorax | A3SS | MXE | 1.72E-03 | 3.42E-04 | 3.09E-03 | 5.01E-03 | ** | * |
| Between event types | Thorax | A3SS | None | 1.16E-03 | -4.88E-05 | 2.36E-03 | 6.86E-02 | ns | * |
| Between event types | Thorax | A5SS | SE | -7.43E-05 | -1.62E-03 | 1.47E-03 | 1.00E+00 | ns | ns |
| Between event types | Thorax | A5SS | IR | -1.61E-03 | -3.62E-03 | 4.02E-04 | 2.02E-01 | ns | * |
| Between event types | Thorax | A5SS | MXE | 1.23E-03 | -1.46E-04 | 2.60E-03 | 1.11E-01 | ns | * |
| Between event types | Thorax | A5SS | None | 6.67E-04 | -5.37E-04 | 1.87E-03 | 6.13E-01 | ns | ns |
| Between event types | Thorax | SE | IR | -1.54E-03 | -3.44E-03 | 3.73E-04 | 1.97E-01 | ns | * |
| Between event types | Thorax | SE | MXE | 1.30E-03 | 8.38E-05 | 2.52E-03 | 2.81E-02 | * | * |
| Between event types | Thorax | SE | None | 7.41E-04 | -2.82E-04 | 1.76E-03 | 3.05E-01 | ns | * |
| Between event types | Thorax | IR | MXE | 2.84E-03 | 1.06E-03 | 4.61E-03 | 7.88E-05 | **** | * |
| Between event types | Thorax | IR | None | 2.28E-03 | 6.28E-04 | 3.92E-03 | 1.17E-03 | ** | * |
| Between event types | Thorax | MXE | None | -5.61E-04 | -1.31E-03 | 1.86E-04 | 2.66E-01 | ns | * |

Supplementary Table 9. Dunn’s test multiple comparisons (two-sided) of nucleotide divergence (ω) between groups of genes (G1 and G2) containing splice events identified by rMATS. Divergence was compared between genes without splicing, those with nonsignificant splice events and those with significantly different splice events between seasons. Divergence was also compared among spliced genes containing different splice events: A3SS, A5SS, SE, IR, and MXE.

| **Analysis** | **Tissue** | **G 1** | **G 2** | **N 1** | **N 2** | **Statistic** | **P-value** | **Adj. P-value** | **Sig.** | **Hyp. test** |
| --- | --- | --- | --- | --- | --- | --- | --- | --- | --- | --- |
| Between None, AS and DS genes | Abdomen | None | AS | 3542 | 2335 | 1.60 | 1.09E-01 | 1.09E-01 | ns | ns |
| Between None, AS and DS genes | Abdomen | None | DS | 3542 | 512 | 3.51 | 4.49E-04 | 1.35E-03 | ** | * |
| Between None, AS and DS genes | Abdomen | AS | DS | 2335 | 512 | 2.52 | 1.16E-02 | 1.74E-02 | * | * |
| Between None, AS and DS genes | Thorax | None | AS | 3465 | 1360 | 0.64 | 5.25E-01 | 5.25E-01 | ns | ns |
| Between None, AS and DS genes | Thorax | None | DS | 3465 | 318 | 1.78 | 7.46E-02 | 2.24E-01 | **ns** | ***** |
| Between None, AS and DS genes | Thorax | AS | DS | 1360 | 318 | 1.35 | 1.77E-01 | 2.65E-01 | **ns** | ***** |
| Between event types | Abdomen | A3SS | A5SS | 258 | 262 | 1.33 | 1.83E-01 | 3.05E-01 | ns | ns |
| Between event types | Abdomen | A3SS | SE | 258 | 243 | 0.75 | 4.54E-01 | 5.68E-01 | ns | ns |
| Between event types | Abdomen | A3SS | IR | 258 | 160 | -1.63 | 1.02E-01 | 1.92E-01 | ns | ns |
| Between event types | Abdomen | A3SS | MXE | 258 | 618 | 2.06 | 3.95E-02 | 1.10E-01 | **ns** | ***** |
| Between event types | Abdomen | A3SS | None | 258 | 3542 | -0.02 | 9.81E-01 | 9.81E-01 | ns | ns |
| Between event types | Abdomen | A5SS | SE | 262 | 243 | -0.56 | 5.75E-01 | 6.64E-01 | ns | ns |
| Between event types | Abdomen | A5SS | IR | 262 | 160 | -2.80 | 5.06E-03 | 2.53E-02 | * | * |
| Between event types | Abdomen | A5SS | MXE | 262 | 618 | 0.49 | 6.27E-01 | 6.72E-01 | ns | ns |
| Between event types | Abdomen | A5SS | None | 262 | 3542 | -1.85 | 6.45E-02 | 1.38E-01 | ns | ns |
| Between event types | Abdomen | SE | IR | 243 | 160 | -2.27 | 2.30E-02 | 8.64E-02 | **ns** | ***** |
| Between event types | Abdomen | SE | MXE | 243 | 618 | 1.13 | 2.58E-01 | 3.87E-01 | ns | ns |
| Between event types | Abdomen | SE | None | 243 | 3542 | -1.03 | 3.02E-01 | 4.11E-01 | **ns** | ***** |
| Between event types | Abdomen | IR | MXE | 160 | 618 | 3.57 | 3.51E-04 | 3.04E-03 | ** | * |
| Between event types | Abdomen | IR | None | 160 | 3542 | 2.02 | 4.39E-02 | 1.10E-01 | ns | ns |
| Between event types | Abdomen | MXE | None | 618 | 3542 | -3.54 | 4.05E-04 | 3.04E-03 | ** | * |
| Between event types | Thorax | A3SS | A5SS | 149 | 149 | 0.27 | 7.84E-01 | 7.84E-01 | ns | ns |
| Between event types | Thorax | A3SS | SE | 149 | 210 | -0.29 | 7.72E-01 | 7.84E-01 | ns | ns |
| Between event types | Thorax | A3SS | IR | 149 | 78 | -1.42 | 1.55E-01 | 5.16E-01 | ns | ns |
| Between event types | Thorax | A3SS | MXE | 149 | 417 | 0.72 | 4.74E-01 | 7.43E-01 | ns | ns |
| Between event types | Thorax | A3SS | None | 149 | 3465 | -0.68 | 4.95E-01 | 7.43E-01 | ns | ns |
| Between event types | Thorax | A5SS | SE | 149 | 210 | -0.59 | 5.58E-01 | 7.61E-01 | ns | ns |
| Between event types | Thorax | A5SS | IR | 149 | 78 | -1.65 | 9.94E-02 | 4.97E-01 | ns | ns |
| Between event types | Thorax | A5SS | MXE | 149 | 417 | 0.38 | 7.02E-01 | 7.84E-01 | ns | ns |
| Between event types | Thorax | A5SS | None | 149 | 3465 | -1.06 | 2.89E-01 | 5.41E-01 | ns | ns |
| Between event types | Thorax | SE | IR | 210 | 78 | -1.26 | 2.06E-01 | 5.16E-01 | ns | ns |
| Between event types | Thorax | SE | MXE | 210 | 417 | 1.17 | 2.41E-01 | 5.16E-01 | ns | ns |
| Between event types | Thorax | SE | None | 210 | 3465 | -0.37 | 7.14E-01 | 7.84E-01 | ns | ns |
| Between event types | Thorax | IR | MXE | 78 | 417 | 2.16 | 3.05E-02 | 2.29E-01 | ns | ns |
| Between event types | Thorax | IR | None | 78 | 3465 | 1.24 | 2.16E-01 | 5.16E-01 | ns | ns |
| Between event types | Thorax | MXE | None | 417 | 3465 | -2.42 | 1.56E-02 | 2.29E-01 | ns | ns |

# Supplementary Figures

## Supplementary Fig. 1


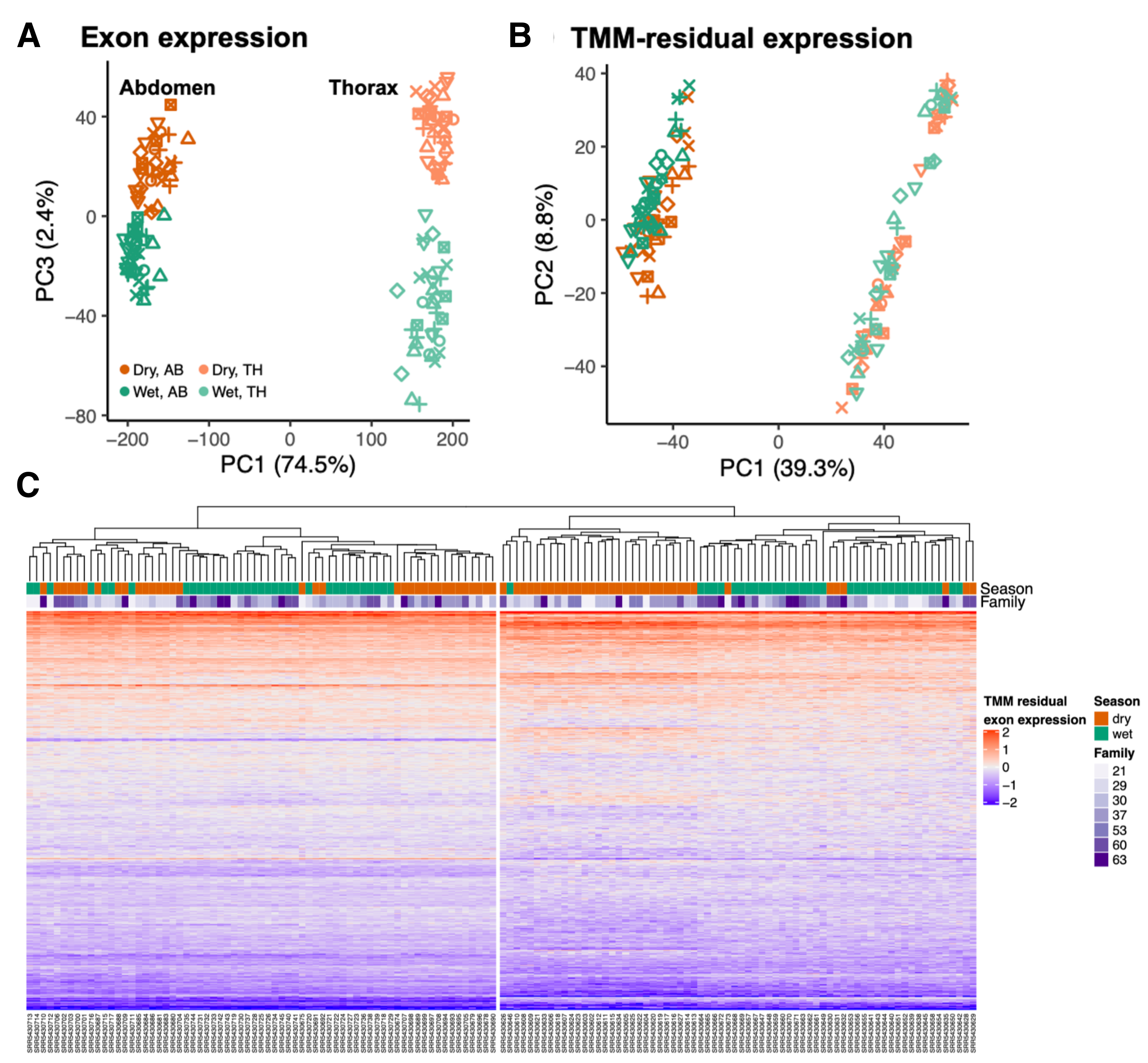


**Supplementary Fig. 1. Differential exon expression between tissues.** (A) PCA of normalized exon expression and (B) residual exon expression (corrected for average exon expression). (C) Normalized residual expression of the top 5000 exons that were differentially expressed in spliced genes between the abdomen and the thorax.

## Supplementary Fig. 2


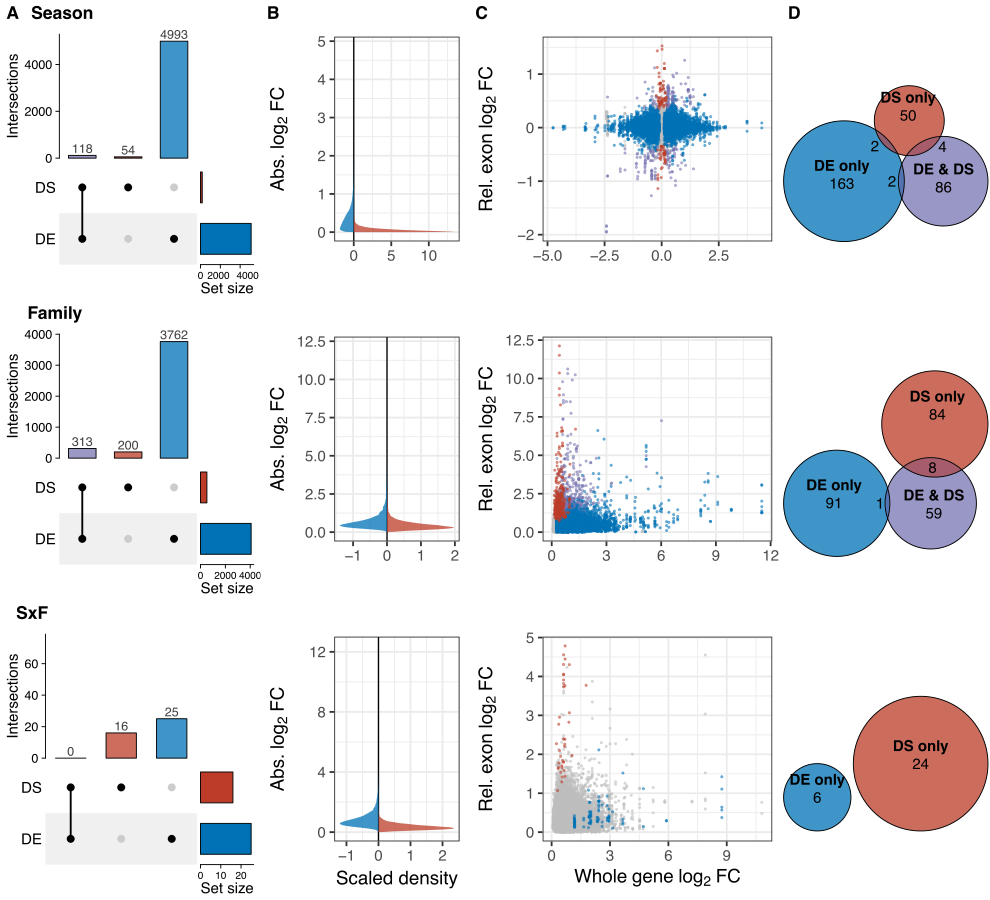


**Supplementary Fig. 2. Overlap between differential splicing (exon expression) and differential expression in the thorax.** (A) Pairwise overlap (purple) of differentially spliced (DS) and differentially expressed (DE) gene sets for the main effects of season and family. SxF genes did not overlap. (B) Scaled densities of the absolute values of whole gene log fold change and exon log fold change (relative to the average fold change of exons within the gene). For seasonal comparisons, fold change represents the change in expression from dry to wet. For family comparisons and SxF interaction, we used the maximum absolute fold change among all families as a proxy for fold change. (C) There was no relationship whole gene log fold change and exon log fold changes when compared between season, among families or SxF. (D) Euler diagrams show that very few gene ontology terms enriched for genes that are differentially expressed (blue), both differentially expressed and differentially spliced (purple), or only differentially spliced (red) overlapped between these gene sets. Circle sizes scale with the number of enriched GO terms (two-sided parentChild Fisher’s Exact Tests, p-value < 0.05), and in cases where the number of shared terms is very small, the number has been placed adjacent to the intersection.

## Supplementary Fig. 3

Figure continues on following page

Figure continues on following page

**Supplementary Fig. 3. Enriched biological process GO terms in the abdomen** were largely not shared among sets of genes that were differentially expressed (DE, blue), both differentially expressed and spliced (DEDS, purple), or differentially spliced (DS, red) (A) between seasonal morphs, (B) among families, or (C) with a season-by-family. Across all gene sets, GO terms were clustered based on semantic similarity (numbers on left, n = 127 clusters) and GO terms falling in the same cluster are considered to be more functionally similar than terms in different clusters. Tile hue corresponds with the degree of enrichment [two-sided Fisher’s Exact Tests, parentChild algorithm; darker = higher -log_10_(p-value)]. Rows are labeled by GO term with truncated descriptions. Full descriptions can be found in Supplementary Data 4.

## Supplementary Fig. 4

Figure continues on following page

**Supplementary Fig. 4. Enriched biological process GO terms in the thorax** were rarely shared among sets of genes differentially expressed (DE, blue), both differentially expressed and spliced (DEDS, purple), or differentially spliced (DS, red) (A) between seasonal morphs, (B) among families, or (C) with a season-by-family interaction. Terms were clustered based on semantic similarity (numbers on left, n = 127 clusters) and terms falling in the same cluster are considered to be more functionally similar than terms in different clusters. Tile hue corresponds with the degree of enrichment [two-sided Fisher’s Exact Tests, parentChild algorithm; darker = higher -log_10_(p-value)]. Rows are labeled by GO term with truncated descriptions. Full descriptions can be found in Supplementary Data 4.

## Supplementary Fig. 5

**Supplementary Fig. 5. Enriched molecular function GO terms in the abdomen** were largely not shared among sets of genes that were differentially expressed (DE, blue), both differentially expressed and spliced (DEDS, purple), or differentially spliced (DS, red) (A) between seasonal morphs, (B) among families, or (C) with a season-by-family interaction. Across all gene sets, GO terms were clustered based on semantic similarity (numbers on left, n = 13 clusters) and GO terms falling in the same cluster are considered to be more functionally similar than terms in different clusters. Tile hue corresponds with the degree of enrichment [two-sided Fisher’s Exact Tests, parentChild algorithm; darker = higher -log_10_(p-value)]. Rows are labeled by GO term with truncated descriptions. Full descriptions can be found in Supplementary Data 4.

## Supplementary Fig. 6

**Supplementary Fig. 6. Enriched molecular function GO terms in the thorax** were largely not shared among sets of genes that were differentially expressed (DE, blue), both differentially expressed and spliced (DEDS, purple), or differentially spliced (DS, red) (A) between seasonal morphs, (B) among families, or (C) with a season-by-family interaction. Across all gene sets, GO terms were clustered based on semantic similarity (numbers on left, n = 13 clusters) and GO terms falling in the same cluster are considered to be more functionally similar than terms in different clusters. Tile hue corresponds with the degree of enrichment [two-sided Fisher’s Exact Tests, parentChild algorithm; darker = higher -log_10_(p-value)]. Rows are labeled by GO term with truncated descriptions. Full descriptions can be found in Supplementary Data 4.

## Supplementary Fig. 7


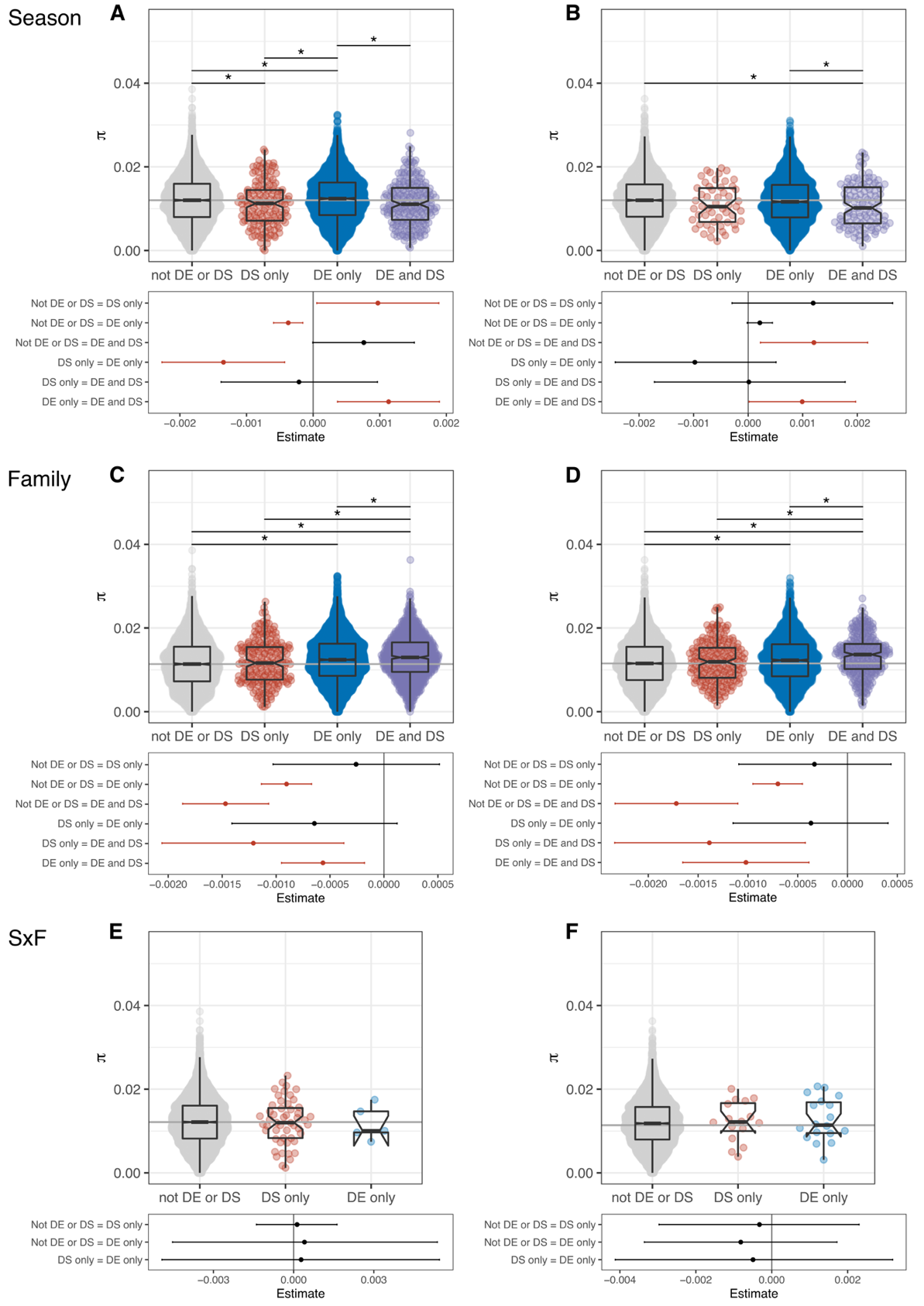


**Supplementary Fig. 7. Nucleotide diversity (π) differences among DS and DE genes** between seasons, among families and among season-by-family comparisons in (A,C,E) the abdomen and (B,D,F) the thorax. Point clouds represent *π* values for each gene set, with sample sizes from left to right: (A) 5588, 146, 4208, 213; (B) 5497, 53, 3828, 118; (C) 3467, 203, 5542, 943; (D) 5366, 199, 2725, 306; (E) 10099, 51, 5; (F) 8562, 16, 18.Values are summarized with boxplots: the center line represents the median, the box encloses the 25^th^-75^th^ quartiles and is notched (median +/- 1.58 * interquartile / $\sqrt{n}$), and whiskers extend to 1.5x the interquartile range. The grey horizontal line indicates the median of genes that were not DE or DS. Lower panels show results of hypothesis tests based on Bayesian linear models, including point estimates and 95% credible intervals (error bars). Statistically meaningful differences do not overlap 0, are highlighted in red, and correspond with asterisks (*) in upper panels A-D. Panels A and C appear in Fig. 3 in the main text.

## Supplementary Fig. 8


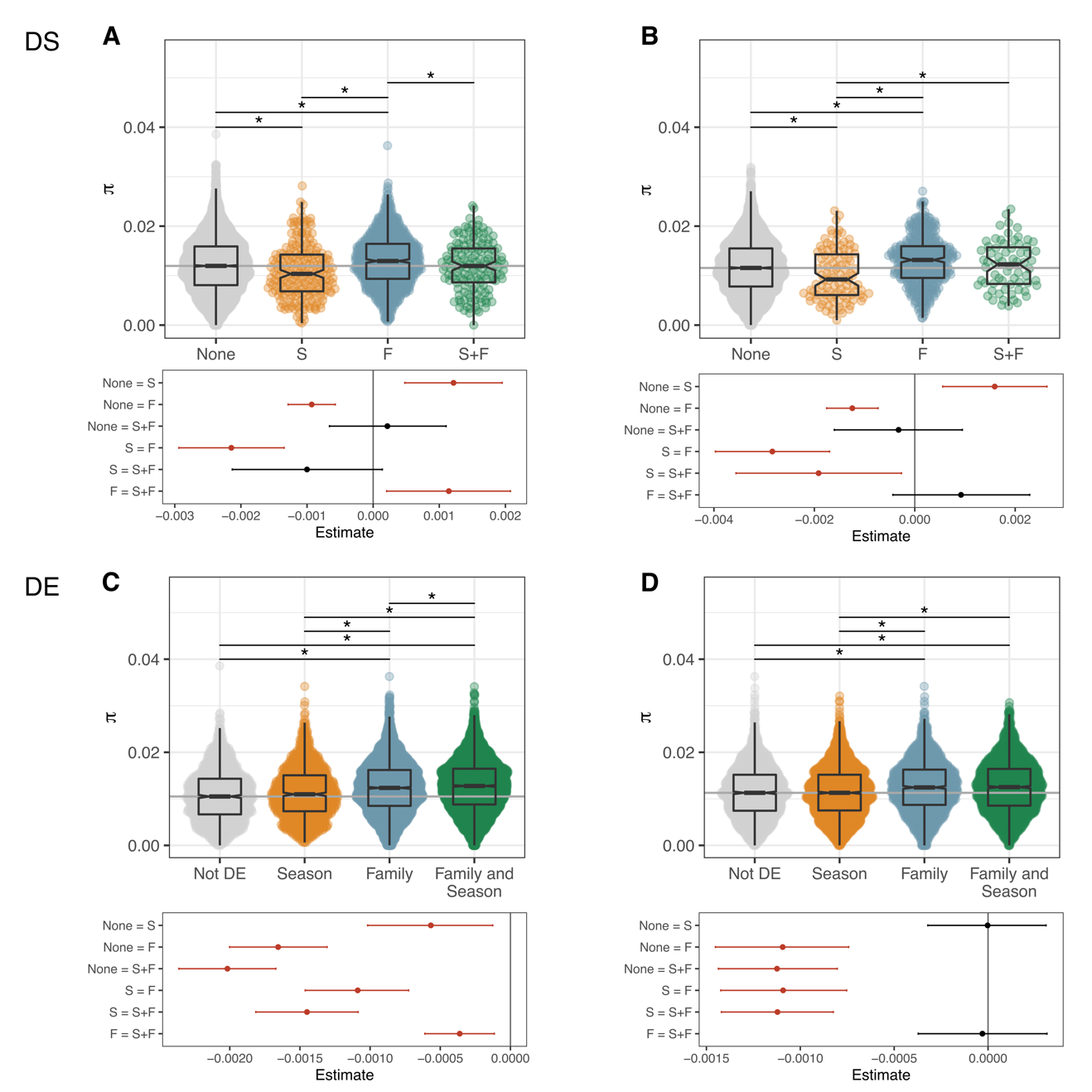


**Supplementary Fig. 8. Nucleotide diversity (π) differences among season and family effects** for both differentially spliced (DS) and differentially expressed (DE) genes in (A,C) the abdomen and (B,D) the thorax. Point clouds represent *π* values for each gene set, with sample sizes from left to right: (A) 7255, 213, 1000, 149; (B) 5934, 104, 438, 67; (C) 1332, 1118, 3672, 4033; (D) 2016, 2629, 1584, 2367. Values are summarized with boxplots: the center line represents the median, the box encloses the 25^th^-75^th^ quartiles and is notched (median +/- 1.58 * interquartile / $\sqrt{n}$), and whiskers extend to 1.5x the interquartile range. The grey horizontal line indicates the median of genes that were not DE or DS. Lower panels show results of hypothesis tests based on Bayesian linear models, including point estimates and 95% credible intervals (error bars). Statistically meaningful differences do not overlap 0, are highlighted in red, and correspond with asterisks (*) in upper panels.

## Supplementary Fig. 9


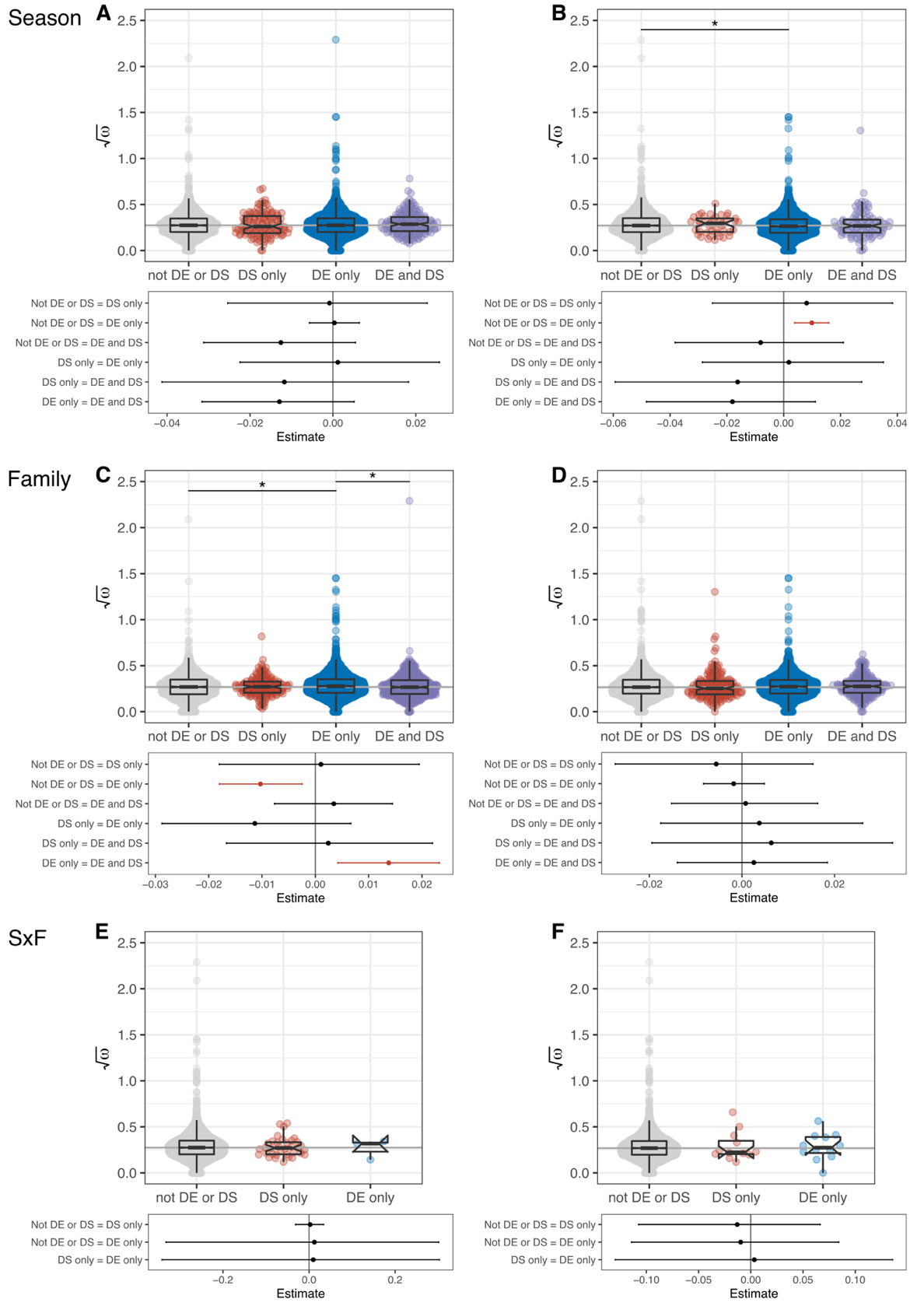


**Supplementary Fig. 9. Nucleotide divergence (ω) differences among DS and DE genes** between seasons, among families and among season-by-family comparisons in (A, C, E) the abdomen and (B, D, F) the thorax.. Point clouds represent $\sqrt{\omega}$ values for each gene set, with sample sizes from left to right: (A) 2955, 113, 3140, 161; (B) 3274, 41, 3045, 96; (C) 1492, 148, 4115, 614 (D) 4013, 158, 2062, 223 (E) 6331, 35, 3, (F) 642, 12, 12. Values are summarized with boxplots: the center line represents the median, the box encloses the 25^th^-75^th^ quartiles and is notched (median +/- 1.58 * interquartile / $\sqrt{n}$), and whiskers extend to 1.5x the interquartile range. The grey horizontal line indicates the median of genes that were not DE or DS. Lower panels show results of hypothesis tests based on Bayesian linear models, including point estimates and 95% credible intervals (error bars). Statistically meaningful differences do not overlap 0, are highlighted in red, and correspond with asterisks (*) in upper panels B and C. Panels A and C appear in Fig. 3 in the main text.

## Supplementary Fig. 10

**Supplementary Fig. 10. Alternative splice event types** in the (A) abdomen and (B) thorax. Small subsets of detected splice events were significantly differentially spliced (colored bars) between seasonal morphs in the abdomen. The panel to the right zooms out to show all detected events (white bars outlined in black). (C, D) Upset plot show the overlap of differentially expressed (DE) and differentially spliced (DS) genes identified by edgeR, and genes containing differentially spliced events identified by rMATS.

## Supplementary Fig. 11

**Supplementary Fig. 11. Nucleotide diversity (π) and divergence (ω) differences among genes** with no splice events, genes with nonsignificant alternative splice events (AS) and genes that contained at least one DS event in the abdomen (A,C) and the thorax (B,D). Point clouds represent *π* or $\sqrt{\omega}$ values for each gene set, with sample sizes from left to right: (A) 4929, 3078, 654; (B) 4532, 1669, 390; (C) 3543, 2336, 512; (D) 3468, 1360, 318. Values are summarized with boxplots: the center line represents the median, the box encloses the 25^th^-75^th^ quartiles and is notched (median +/- 1.58 * interquartile / $\sqrt{n}$), and whiskers extend to 1.5x the interquartile range. The grey horizontal line indicates the median of genes that were not spliced. Lower panels show results of hypothesis tests based on Bayesian linear models, including point estimates and 95% credible intervals (error bars). Statistically meaningful differences do not overlap 0, are highlighted in red, and correspond with asterisks in upper panels. Panel A appears in Fig. 4 in the main text.

## Supplementary Fig. 12

**Supplementary Fig. 12. Nucleotide diversity (π) and divergence (ω) differences among event-based DS and DE genes** between seasons in (A,C) the abdomen and (B,D) the thorax. Point clouds represent *π* or $\sqrt{\omega}$ values for each gene set, with sample sizes from left to right: (A) 4731, 273, 4770, 381; (B) 3462, 138, 4744, 252; (C) 3301, 216, 3474, 296; (D) 2500, 111, 3635, 207. Values are summarized with boxplots: the center line represents the median, the box encloses the 25^th^-75^th^ quartiles and is notched (median +/- 1.58 * interquartile / $\sqrt{n}$), and whiskers extend to 1.5x the interquartile range. The grey horizontal line indicates the median of genes that were not DE or DS. Lower panels show results of hypothesis tests based on Bayesian linear models, including point estimates and 95% credible intervals (error bars). Statistically meaningful differences do not overlap 0, are highlighted in red, and correspond with asterisks in upper panels.

## Supplementary Fig. 13

**Supplementary Fig. 13. Nucleotide diversity (π) and divergence (ω) differences among AS and DS genes for each splice event type** in the abdomen (A,C) and the thorax (B,D). Point clouds represent *π* or $\sqrt{\omega}$ values for each gene set, with sample sizes from left to right: (A) 1028, 98, 1035, 145, 937, 332, 727, 68, 1898, 200; (B) 474, 73, 519, 85, 572, 219, 342, 36, 989, 109; (C) 813, 78, 807, 114, 688, 262, 573, 50, 1405, 145; (D) 395, 59, 428, 70, 459, 178, 279, 31, 800, 86;. Values are summarized with boxplots: the center line represents the median, the box encloses the 25^th^-75^th^ quartiles and is notched (median +/- 1.58 * interquartile / $\sqrt{n}$), and whiskers extend to 1.5x the interquartile range. The grey horizontal line indicates the median of genes that were not spliced. An asterisk (*) centred over a horizontal line between groups indicates a meaningful statistical difference between these groups whereas an asterisk centred over the DS points indicates a statistical difference from the grey line. Lower panels show results of hypothesis tests based on Bayesian linear models, including point estimates and 95% credible intervals (error bars). Statistically meaningful differences do not overlap 0, are highlighted in red, and correspond with asterisks in upper panels. Panel A appears in Fig. 4 in the main text.

## Supplementary Fig. 14

**Supplementary Fig. 14. Normalized read coverage of annotated genes in the abdomen (n = 69) and thorax (n = 70).** (A) Normalized read coverage across transcripts shows no evidence of degraded RNA, which would result in a 3’ coverage bias. (B) Rather, reads tended to show a 5’ bias, and this bias differed between seasons in the abdomen but not the thorax (two-sided t-tests).

# Supplementary Notes

## Supplementary Note 1.

All source data and R code necessary to reproduce figures in the main text can be found in the Source Data file, SourceData_B_anynana_AS.zip. This file includes two Rmarkdown documents:

- **B_anynana_AS.Rmd:** This markdown document loads data from the ‘Data_in’ folder, including population genetic parameters (pi, theta, omega) and output from previous differential splicing and differential expression analyses. The code in this markdown file can be used to recreate all main text figures (panels showing data only) and most of the supplementary figures, excepting Supplementary Fig. 1 and 12. The PCAs shown in figure 1 in the main text are also exceptions and the necessary code is included on the github site.
- **B_anynana_AS_NatCommR1_additionalfigs.Rmd:** This markdown document includes the code necessary to perform the analyses and make the plots shown in Supplementary Fig. 12, and to create the map in Fig. 3A.

Many of the data frames used as input for the above Source Data were generated from RNAseq data available from from NCBI archives (PRJNA376691) using code available on the public github site <https://github.com/rstewa03/B_anynana_differentialSplicing>. This publicly available code can also be used to generate the remaining supplementary figures, including PCAs and heatmaps shown in Fig. 1 and Supplementary Fig. 1.
