# Supplementary material for: Alternative splicing in seasonal plasticity and the potential for adaptation to environmental change
Source: Nat Commun. 2022 Feb 8;13:755. doi: 10.1038/s41467-022-28306-8 (PMC8825856; doi:10.1038/s41467-022-28306-8)
Supplement: Supplementary file 2 — Reporting Summary [file 41467_2022_28306_MOESM2_ESM.pdf]

nature research

Corresponding author(s): Rachel A Steward

Last updated by author(s): Dec 8, 2021

Reporting Summary

Nature Research wishes to improve the reproducibility of the work that we publish. This form provides structure for consistency and transparency in reporting. For further information on Nature Research policies, see our Editorial Policies and the Editorial Policy Checklist.

Statistics

For all statistical analyses, confirm that the following items are present in the figure legend, table legend, main text, or Methods section.

n/a	Confirmed	
[ ]	[x]	The exact sample size (*n*) for each experimental group/condition, given as a discrete number and unit of measurement
[ ]	[x]	A statement on whether measurements were taken from distinct samples or whether the same sample was measured repeatedly
[ ]	[x]	The statistical test(s) used AND whether they are one- or two-sided<br>*Only common tests should be described solely by name; describe more complex techniques in the Methods section.*
[ ]	[x]	A description of all covariates tested
[ ]	[x]	A description of any assumptions or corrections, such as tests of normality and adjustment for multiple comparisons
[ ]	[x]	A full description of the statistical parameters including central tendency (e.g. means) or other basic estimates (e.g. regression coefficient) AND variation (e.g. standard deviation) or associated estimates of uncertainty (e.g. confidence intervals)
[ ]	[x]	For null hypothesis testing, the test statistic (e.g. *F*, *t*, *r*) with confidence intervals, effect sizes, degrees of freedom and *P* value noted<br>*Give P values as exact values whenever suitable.*
[ ]	[x]	For Bayesian analysis, information on the choice of priors and Markov chain Monte Carlo settings
[x]	[ ]	For hierarchical and complex designs, identification of the appropriate level for tests and full reporting of outcomes
[x]	[ ]	Estimates of effect sizes (e.g. Cohen's *d*, Pearson's *r*), indicating how they were calculated

Our web collection on statistics for biologists contains articles on many of the points above.

Software and code

Policy information about availability of computer code

Data collection

RNAseq reads were filtered iand trimmed using bbmap_35.69 (BBDuk2)
Reads were mapped with STAR (v.2.5.0)
Mapped reads were sorted and indexed with Samtools (v.1.9)
RNA degradation was assessed with Picard tools (v. 1.139)
Raw WGS reads were trimmed with Cutadapt (v.1.21) and Sicle (v.1.2).
Trimmed reads were mapped bwa-mem (v.0.7.15-r1140)
Duplicated reads were removed using Picard tools (v. 2.0.1)
Polymorphism was computed with angsd (v. 0.931-10-g09a0fc5) and processed in R (v.3.6.3) to calculate a nucleotide diversity value for the coding sequence of each gene.
Genomes were aligned with macse (v.1.01b)

Data analysis

Mapped RNAseq reads were quantified with Rsubread (v. 2.0.0) featureCounts.
Differential splicing and expression were analysed in R (v. 4.0.0) with edgeR (v. 3.30.3), supported by
- tidyverse (v. 1.3.1), as
- ggpubr (v. 0.4.0)
- ggsci (v. 2.9)
- GeneOverlap (v. 1.24.0)
- ComplexHeatmap (v. 2.4.3)
- stats (v. 4.0.0)

We generated a gene ontology annotation using EggNOG (v. 50)

We analysed gene set enrichment with topGO (v.2.28) and REVIGO (http://revigo.irb.hr/)
In addition to previously listed packages, gene set enrichment was visualised in R (v. 4.0.0) with the support of
- eulerr (v. 6.1.1)
- simplifyEnrichment (v. 3.13)

Statistical analyses were performed in R using brms (v. 2.14.4) and rstatix (v. 0.7.0)
All code has been provided with the paper in the Source Data file or can be accessed at https://github.com/rstewa03/B_anynana_differentialSplicing, DOI: 10.5281/zenodo.5762211

For manuscripts utilizing custom algorithms or software that are central to the research but not yet described in published literature, software must be made available to editors and reviewers. We strongly encourage code deposition in a community repository (e.g. GitHub). See the Nature Research guidelines for submitting code & software for further information.

Data

Policy information about availability of data

All manuscripts must include a data availability statement. This statement should provide the following information, where applicable:
- Accession codes, unique identifiers, or web links for publicly available datasets
- A list of figures that have associated raw data
- A description of any restrictions on data availability

Bicyclus anynana RNA-seq data used to estimate differential splicing and differential expression in this study were accessed from NCBI archives (PRJNA376691, https://www.ncbi.nlm.nih.gov/bioproject/376691). We accessed the B. anynana genome v1.2 from NCBI, PRJNA434100, (https://www.ncbi.nlm.nih.gov/genome/10970?genome_assembly_id=358767). Illumina short-read whole genome data generated in this study and used to estimate population genetic parameters were archived at NCBI under accession number PRJNA786886 (https://www.ncbi.nlm.nih.gov/bioproject/786886). The genome of Pararge aegeria that was used to identify single copy orthologs and calculate nucleotide divergence was accessed from NCBI, PRJEB28004, https://www.ncbi.nlm.nih.gov/assembly/GCA_900499025.1/) . Metadata and results of differential expression and spicing analyses have been included in the supplementary data file, as described in the supplementary information file. Source data necessary to perform subsequent analyses are provided in the SourceData_B_anynana_AS.zip file, as described in supplementary note 1 in the supplementary information file.

Field-specific reporting

Please select the one below that is the best fit for your research. If you are not sure, read the appropriate sections before making your selection.

☐ Life sciences ☐ Behavioural & social sciences ☒ Ecological, evolutionary & environmental sciences

For a reference copy of the document with all sections, see nature.com/documents/nr-reporting-summary-flat.pdf

Ecological, evolutionary & environmental sciences study design

All studies must disclose on these points even when the disclosure is negative.

Study description	The study analyzed differences in whole gene expression, exon expression and splice events among lab-reared Bicyclus anynana butterflies using archived RNAseq data, whole genome resequencing data from wild butterflies, and a comparison of published genomes. Butterflies from seven families were reared in a split-brood design in two different temperature environments, one representing the dry season (19C) and one representing the wet season (27C), allowing for a fully factorial comparison of environment (season) and genotype (family). We further compared nucleotide diversity and divergence among differentially expressed and differentially spliced genes.
Research sample	RNAseq data was accessed from NCBI PRJNA376691, first published by Oostra et al. (2018). The original samples were taken from the thorax and abdomen of outbred lab-reared Bicyclus anynana butterflies. All individuals (n = 70) were adult females and were sacrificed one day after eclosion. Females were reared in two temperature treatments (19C and 27C).<br>Whole genome sequencing (WGS) data was generated for wild-caught adult female B. anynana butterflies (n = 5) from a population near Zomba, Malawi.<br><br>Oostra, V., Saastamoinen, M., Zwaan, B. J. & Wheat, C. W. Strong phenotypic plasticity limits potential for evolutionary responses to climate change. Nat Commun 9, 1–11 (2018).
Sampling strategy	The five individuals for WGS were selected randomly from among wild sampled individuals and are sufficient to evaluate nucleotide diversity in the population.<br>We used all available archived RNAseq samples from NCBI PRJNA376691. For more details on sampling strategy, see Oostra et al. 2018. Briefly, abdomen and thorax samples were collected from up to six individuals in each season for each of the seven families in order to maximize the number of families while maintaining sufficient replication within families. Several samples were excluded due to poor quality. Here, we used all sequences that were archived from the previous study.<br><br>Oostra, V., Saastamoinen, M., Zwaan, B. J. & Wheat, C. W. Strong phenotypic plasticity limits potential for evolutionary responses to climate change. Nat Commun 9, 1–11 (2018).
Data collection	RNAseq data were accessed from National Centre for Biotechnology Information (NCBI) Sequence Read Archive (SRA) BioProject ID

	PRJNA376691. More details on data collection can be found in Oostra et al. 2018. WGS data were generated from five wild individuals collected from Zomba, Malawi, by MA de Jong.<br><br>Oostra, V., Saastamoinen, M., Zwaan, B. J. & Wheat, C. W. Strong phenotypic plasticity limits potential for evolutionary responses to climate change. Nat Commun 9, 1–11 (2018).
Timing and spatial scale	Details on archived RNAseq samples can be found in Oostra et al. 2018. Briefly, the lab reared individuals came from a single outbred population. All caterpillars were reared at the same time, regardless of treatment.<br>The wild caught individuals were sampled as part of a larger phylogeographic study were collected from the same sampling location at the same time in March 2007 and are sufficient to evaluate nucleotide diversity in the population at that time.<br><br>Oostra, V., Saastamoinen, M., Zwaan, B. J. & Wheat, C. W. Strong phenotypic plasticity limits potential for evolutionary responses to climate change. Nat Commun 9, 1–11 (2018).
Data exclusions	No data were excluded from the analyses, except those already excluded in previous studies. Two extreme outlier values for nucleotide divergence were excluded from statistical analyses to avoid biasing the analyses.
Reproducibility	Analyses were performed separately in both tissues, the abdomen and thorax, to evaluate whether patterns of nucleotide divergence and/or diversity were supported in multiple tissues. We repeated the analyses of nucleotide divergence and diversity using two different splicing software to show the overall patterns of reduced nucleotide diversity were reproducible with both approaches.
Randomization	In the previous study, caterpillars within families were randomly assigned to seasonal treatments.
Blinding	Blinding was not relevant to the study, as we were analysing gene expression and splicing patterns in the same way across all replicates, then comparing them between treatments.

Did the study involve field work? ☐ Yes ☒ No

Reporting for specific materials, systems and methods

We require information from authors about some types of materials, experimental systems and methods used in many studies. Here, indicate whether each material, system or method listed is relevant to your study. If you are not sure if a list item applies to your research, read the appropriate section before selecting a response.

Materials & experimental systems

n/a	Involved in the study
☒	☐ Antibodies
☒	☐ Eukaryotic cell lines
☒	☐ Palaeontology and archaeology
☐	☒ Animals and other organisms
☒	☐ Human research participants
☒	☐ Clinical data
☒	☐ Dual use research of concern

Methods

n/a	Involved in the study
☒	☐ ChIP-seq
☒	☐ Flow cytometry
☒	☐ MRI-based neuroimaging

Animals and other organisms

Policy information about studies involving animals; ARRIVE guidelines recommended for reporting animal research

Laboratory animals	RNAseq data was accessed from NCBI PRJNA376691. The original samples were taken from the thorax and abdomen of outbred lab-reared Bicyclus anynana butterflies. All individuals (n = 70) were adult females and were sacrificed one day after eclosion. Females were reared in two temperature treatments (19C and 27C). For more details, see Oostra et al. 2018.<br><br>Oostra, V., Saastamoinen, M., Zwaan, B. J. & Wheat, C. W. Strong phenotypic plasticity limits potential for evolutionary responses to climate change. Nat Commun 9, 1–11 (2018).
Wild animals	We generated whole-genome DNA resequencing data from a wild B. anynana population from Zomba, Malawi (15°22'S, 35°19'E). Butterflies were caught as part of a larger phylogeographic study by MA de Jong. See 'Field-collected samples' for more detail.
Field-collected samples	The Zomba population was collected and brought to the laboratory in March 2007 and has been studied previously in phylogeographic analyses using candidate genes (de Jong et al. 2011, 2013). Butterflies were frozen alive at -80C and stored at that temperature, excepting short periods of freezer malfunction.<br><br>de Jong, M. A., Wahlberg, N., Eijk, M. van, Brakefield, P. M. & Zwaan, B. J. Mitochondrial DNA Signature for Range-Wide Populations of Bicyclus anynana Suggests a Rapid Expansion from Recent Refugia. PLOS ONE 6, e21385 (2011).<br><br>de Jong, M. A., Collins, S., Beldade, P., Brakefield, P. M. & Zwaan, B. J. Footprints of selection in wild populations of Bicyclus anynana along a latitudinal cline. Mol Ecol 22, 341–353 (2013).

Ethics oversight	The study did not require any ethical approval.

Note that full information on the approval of the study protocol must also be provided in the manuscript.
