# Supplementary material for: Alternative splicing in seasonal plasticity and the potential for adaptation to environmental change
Source: Nat Commun. 2022 Feb 8;13:755. doi: 10.1038/s41467-022-28306-8 (PMC8825856; doi:10.1038/s41467-022-28306-8)
Supplement: Supplementary file 4 — Description of Additional Supplementary Files [file 41467_2022_28306_MOESM4_ESM.pdf]

Title: Supplementary Data 1.
Description: Sample metadata, adapter trimming (columns beginning with 'AdaptTrim'), quality filtering (columns beginning with 'QualFilt'), alignment with STAR (columns beginning with Mapping) and feature assignment (columns beginning with FeatureCount) statistics.

Title: Supplementary Data 2.
Description: Differentially spliced (DS) genes [Simes correction of quasi-likelihood F-tests; BH adjusted p-values (FDR) < 0.05] by season, family and season x family interaction detected by edgeR in the abdomen and thorax. Exon expression was contrasted between seasonal morphs, resulting in a single p-value per locus. Family and season-by-family (SxF) interactions were assessed in a series of pairwise comparisons between groups, with a total of 21 contrasts for each. Both the minimum FDR and the number of pairwise contrasts with FDR < 0.05 are reported.

Title: Supplementary Data 3.
Description: Differentially expressed genes between seasons, among families and among season x family interaction (SxF) by edgeR in the abdomen and thorax. Benjamin-Hochberg adjusted p-values (FDR) are reported for the main effects and interaction terms of quasi-likelihood F-tests for each gene.

Title: Supplementary Data 4.
Description: Enriched GO terms (parent-child Fisher's exact tests (two-sided) p-value <0.05) were clustered based on semantic similarity (simplifyEnrichment).

Title: Supplementary Data 5.
Description: Differentially spliced (DS) events between seasonal morphs detected by rMATS in the abdomen and thorax.
